# Modeling the effects of farming management practices on soil organic carbon stock under two tillage practices in a semi-arid region, Morocco

**DOI:** 10.1016/j.heliyon.2020.e05889

**Published:** 2021-01-02

**Authors:** Ibtissame Lembaid, Rachid Moussadek, Rachid Mrabet, Ahmed Douaik, Ahmed Bouhaouss

**Affiliations:** a Faculty of Sciences, Mohammed V University in Rabat, Morocco; b National Institute for Agricultural Research of Rabat (INRA), Morocco

**Keywords:** Soil organic carbon, DNDC model, Farming management practices, No-tillage system, Climate change

## Abstract

Farming management practices are of paramount importance for soil organic carbon (SOC) sequestration in carbon (C) cycling at different scales. However, due to a lack of proper methodologies, estimating the impacts of different soil management practices on overall SOC stock remains inadequately quantified. In this paper, a process-based model, Denitrification-Decomposition (DNDC), was validated on midterm (9 years) and employed depending on the local climate, soil and management conditions, to assess the impacts of alternative management practices on SOC stock under two tillage systems, in a semi-arid region of Morocco. Validated results showed a good agreement between model simulated and observed values, based on the normalized root mean square error (RMSE) and Pearson correlation coefficient (r). This agreement indicates that the DNDC model could capture patterns and magnitudes changes across the climate zone, soil type, and management practices. Modeled results pointed out that, under no-tillage practice (NT), the SOC content increased by 30% compared to conventional tillage (CT). During the simulated period (9 years), the SOC sequestration potential (CSP) has been greatly improved with increased crop residue rate and application of farmyard manure (FY-manure). This increase ranged from 415 kg C/ha to 1787 kg C/ha under NT practice, and from 150 kg C/ha to 818 kg C/ha under CT system. In contrast, increasing fertilizer rate had low to negligible effect on SOC stock. On the other hand, CSP declined by 107–335 kg C/ha and by 177–354 kg C/ha under NT and CT practices respectively, when decreasing N-fertilizer rates. In light of these results, an increase in crop residue rate returned at surface after harvest and application of organic fertilizer, especially under NT practice, can substantially improve SOC stock in a semi-arid region.

## 1 Introduction

Soil organic carbon (SOC) is considered as a key component in agroecosystems. Soil holds approximately 2344 Gt (1 gigaaton = 1billion tons). The first meter of the soil contains 54% of stored SOC, from which 41% are warehoused in the top 20 cm (Jobbagy and Jackson, 2000; Lackner, 2002). C sequestration has been displayed as an inexpensive approach to relieve greenhouse effect. This process converts the atmospheric carbon dioxide (CO_2_) into biotic or abiotic C stored in agroecosystems (Lackner, 2003). Important decisions need to be made regarding future tolerable levels of atmospheric CO_2_ content, as well as the land and fossil fuel use strategies that will permit us to achieve these goals (Sarmiento and Bender, 1994). SOC pool represents dynamic C equilibrium in terms of benefits and losses (Farahbakhshazad et al., 2008). A small change of SOC may induce a significant change in large-scale C cycling which can add important amounts of biomass to the soil, lead to minimal soil disturbance, preserve soil and water, enhance soil structure, and raise soil biotic activity (Mrabet, 2010a).

In semi-arid areas of Mediterranean basin, the climatic consequences for rain-fed crops are: (i) high mineralization rates of soil organic matter (SOM) augmented by high temperatures during wet periods; (ii) insufficient residues to cover the soil surface after periods of drought, which influence crop production; (iii) some tillage practices are performed immediately after harvesting to bury residues by disking, and even to partially prepare the seedbed in dry conditions; and (iv) cereal crop residues are currently baled after harvesting (Martens et al., 2005; Madejo'n et al., 2007). In Morocco, most soils have a low SOM (<2%). This loss of SOM is estimated about 30 % between 1987 and 1997 (Barbera et al., 2012) due mainly to inappropriate soil management practices, such as CT. During recent years, many studies showed that the tillage practices have been a major contributor to land degradation and soil fertility decrease (Soudi et al., 2003), including compaction of soil below the tillage depth, increased vulnerability to water and wind erosion, and enhanced SOM mineralization. Indeed, the Food and Agriculture Organization (FAO) estimated that 71% of Moroccan agricultural lands are degraded and need to adopt conservation systems and other sustainable land management strategies, whose purpose is to secure food production to meet the growing population needs (Bot and Benites, 2005). Therefore, conservation agriculture (CA) has been adopted in many parts of the world as an alternative approach to increase profits and food security (FAO, 2002), improve the production efficiency and soil productivity, mitigate climate change, protect soil and make agriculture more sustainable (Sangar et al., 2005; Dumanski et al., 2006; Hobbs and Govaerts, 2010; Derpsch et al., 2010, 2011).

Morocco is one of the first emerging countries to adopt CA as an alternative strategy to protect natural resources, especially in the semi-arid areas. Several researchers from Morocco pointed out that NT system enhances the SOC contents (Bessam and Mrabet, 2003), mitigates CO_2_ emission (Moussadek et al., 2011a), reduces runoff and erosion (Dimanche, 1997; Moussadek et al., 2011b; Briak et al., 2016), and increases crop yields (Bouzza., 1990; Mrabet, 2002, 2008, 2010b, 2011). Moreover, several studies have indicated that SOC enhancing practices, which help increasing SOC amount, offer to be low-cost solutions to improve productivity (Kanyenji et al., 2020). Therefore, they have been universally proposed to be a measure of soil quality and mitigating climate change.

SOC enhancing strategies, suggested by soil scientists worldwide, generally involve optimizing alternative management practices through modification of C inputs from crop residue returned at the surface as mulch, and/or application of organic fertilizer, including FY-manure and compost. The crop residue management is an important component of SOC (Li et al., 2003). If returned to the soil, crop residues would constitute a direct C input, and therefore, would perform a double function, facing global warming and food security, through increasing C sequestration in agriculture and improving grain yields (Zhang et al., 2008). Many previous studies have reported that the improvement in SOC stock is usually expected when crop residue returned at the surface is increased (Moebius-Clune et al., 2008; Hammerbeck et al., 2012). Over the recent years, the crop residue management in Morocco has changed significantly by returning until 30% of crop residue under NT system. This approach is considered as one of the most important practices which improves the photosynthetic input of C into the soil, enhances the SOC stock, and greatly sequester C from atmosphere (Follett, 2001; Lal, 2004).

On the other hand, fertilizer inputs may change the relative bioavailability of SOC (El Hassani et al., 2020). Productivity is generally controlled by the nutrient level of soil, especially nitrogen (N) concentration (Czóbel et al., 2013). Before 1970s, organic fertilizers, such as FY-manure and compost, were used as major source of soil nutrients in Morocco. However, these organic inputs were replaced gradually by mineral fertilizers, to ensure local food security, threatened by the rapid growing demand for food driven by the ever increasing population.

During the last 20 years, dynamic modeling is considered as an effective approach to estimate SOC stock and loss from cropland under global warming scenarios. Several biogeochemical models have been designed and developed for this purpose. These models include CANDY (Franko, 1996), ROTHC (Jenkinson et al., 1987), CENTURY (Parton et al., 1996), DAISY (Mueller et al., 1996), DNDC (Li et al., 1992a), and NCSOIL (Molina et al., 1983).

The present paper aims to assess the impact of different farming management practices on SOC stock. The objectives are: i) to evaluate the model performance, and identify the SOC dynamics under baseline scenarios with modelling approach, ii) to use the DNDC model depending on the local climate, soil and management conditions in order to predict the impact of alternative management practices under two tillage practices on SOC stock. Three hypotheses were advanced: i) the SOC stock can evolve differently by increasing crop residue rate returned at surface from the baseline scenarios (30% under NT and, 0% under CT), to 50%, 70% and 90%, ii) the SOC stock will be improved when 500 kg/ha and 1000 kg/ha of FY-manure are added to current management, and iii) the changes in N-fertilize rates from the existing rates (increase and decrease by 30%, 50% and 80%) can influence the SOC stock dynamics over the 9-simulated years.

## 2 Materials and methods

### 2.1 Description of study site

**Figure 1:**
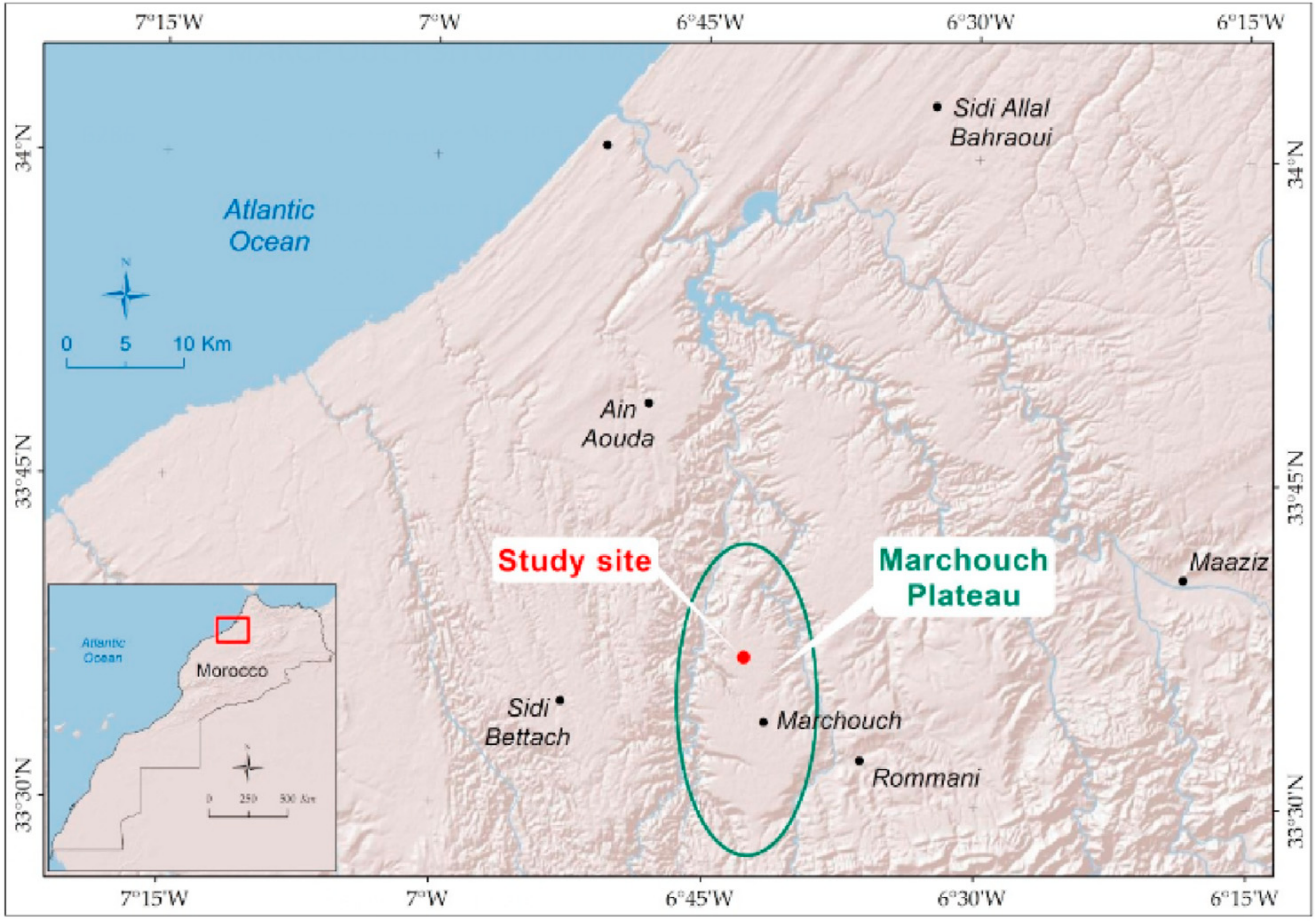
Map showing the location of study site.

**Figure 2:**
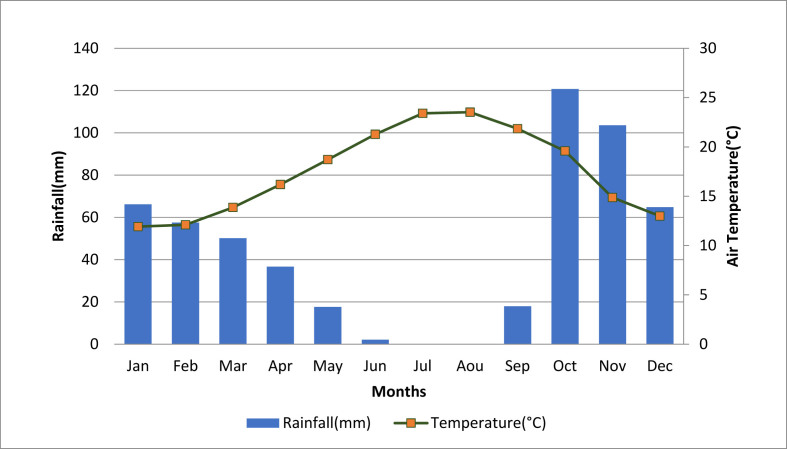
Monthly average of rainfall and temperature during 2008–2016 at Merchouch Station in Morocco.

The experiment was carried out at the experimental station of Merchouch Plateau (33°34′N, 6°42′W, and 425 m elevation), located in a semi-arid zone of Morocco (Figure 1). The annual average precipitations and temperatures are respectively 450 mm and 23 °C. Figure 2 shows the average rainfall and temperature monthly time-series during the simulated years (2008–2016). According to WRB (2006), the experimental site is classified as Vertisol. The soil is rich in clay (>50% clay), poor in SOC content (12.2 g/kg of soil), with a basic pH (Table 1), and poor drainage capacity.

**Table 1:** Location and characteristics of climate, soil and cropping system of selected site.

Site	Location	Temperature (°C),Precipitation (mm)	Cropping system	SOC g/kg of soil	Soil density g/cm^3^	pH	Clay fraction
Merchouch 33°34′N 6°42′W	Province of khemisset	23/450	Winter-wheat/Legumes	12.2	1.4	7.6	50.5

### 2.2 Experimental set-up

This experiment has been conducted since 2004 under two tillage practices on 2 ha each. Conventional tillage (CT) consists on plowing up to 30 cm deep then proceeding to a shallow tillage (10–15 cm) in order to prepare a fine seedbed and to bury plant residues. Unlike CT, the no-tillage method (NT) is a single operation which holds an opening of 2–3 cm from the ground with a special NT drill allowing to put the seeds at 5 cm depth. Winter wheat-legumes rotation and crop management were similar under both CT and NT practices. The trials were installed in mid-November for spring wheat and mid-December for lentil with respective seed rates of 140 and 40 kg ha^−1^. Before sowing, wheat and lentil received a rate of 150 and 100 kg ha^−1^ respectively of a NPK complex fertilizer 14-18-14 (14% N- 28% P2O5 -14% K2O). At the end of February, wheat received 100 kg ha^−1^ of urea. According to the conventional farming practices of the region, all crop residues were removed after harvest from the field under CT, while about 30 % of the crop residues were maintained at the surface under NT practice.

### 2.3 The DNDC model

The Denitrification–Decomposition (DNDC) model was initially developed to predict C and N biogeochemical cycles in the U.S. agroecosystems (Li et al., 1992b, 1994). The model consists of six interacting sub-models, describing the generation, decomposition, transformation of SOM and the outputs of the dynamic components of SOC and greenhouse gas fluxes (Zhao et al., 2014). The six sub-models include:1) a soil climate component using air temperature, precipitation, and soil physical properties, data to calculate soil temperature, moisture, redox potential (Eh) profiles, and soil water fluxes through time; 2) a nitrification component; 3) a denitrification module, which calculates hourly denitrification rates, N_2_O, NO, and N_2_ production; 4) simulation of SOC dynamics and CO_2_ production; 5) a plant growth component, assessing daily root respiration, water, N uptake, and plant growth; and 6) a fermentation component, considering daily methane (CH4) production and oxidation (Li, 2000; Li et al., 2006; Abdalla et al., 2009; Cui et al., 2014; Amponsah et al., 2019; Shi et al., 2020).

### 2.4 Mandatory inputs parameters

The inputs required for the DNDC model include: location (site; latitude), climate (daily mean rainfall and air temperature), soil properties (soil texture; soil bulk density; soil pH; SOC at surface), and farming management practices (crop type; crop rotation; no. crops per year); planting and harvest date; cover crop); fertilizer (type, method, rate, no. of applications, dates, depth); tillage (method, no. of applications, dates)).

### 2.5 Field measurements for DNDC model verification

Model validation against experimental data is essential to verify that the model is correctly simulating the underlying processes (Giltrap et al., 2010). Tonitto et al. (2018) define the methodology for models application to improve their use for quantifying the environmental performance of agricultural systems and managed landscapes. The DNDC model's validation in this study was conducted by following step-by-step instructions detailed by Li (2012).

The measured SOC values combined with other relevant data of the site (climate, soil properties, crop type, rotation, and cropping practices) were collected from the database of the National Institute of Agricultural Research and the Moroccan General Direction of Meteorology. These data were utilized for DNDC validation during 9 years (from 2008 to 2016). Modeled SOC values were compared with observed SOC collected from the literature (Moussadek, 2012; Laghrour et al., 2015, 2016; Moussadek et al., 2014), and the National Institute of Agricultural Research.

Several metrics have been developed to assess the ‘‘goodness of fit’’. Two statistical indicators were commonly used to verify the DNDC model performance and the modeled results acceptability: the root mean square error (RMSE) and the Pearson correlation coefficient (r). During the period 2008–2016, field observations indicated that the mean SOC content under NT system was 30% higher (12.2 g C/kg) than under CT (9,3 g C/kg soil) at the top 20 cm depth of soil (Figure 4).

The Pearson correlation coefficient (Equation (1)) is useful in assessing how well the shape of the simulation matches the shape of the measurement. This coefficient ranges from -1 to 1, with values close to 1 indicating a good agreement (Krause et al., 2005).

(1) $$r=\left[\frac{\sum \left(O_{i}-\bar{O}\right)\left(P_{i}-\bar{P}\right)}{\sqrt{\sum {\left(O_{i}-\bar{O}\right)}^{2}\sum {\left(P_{i}-\bar{P}\right)}^{2}}}\right]$$

The bias in the total difference between simulations and measurements was determined by calculating the RMSE (Equation (2)). RMSE is one of the most widely used statistical indicators which measures the average magnitude of the difference between predictions (P) and observations (O). It ranges from 0 (good model performance) to positive infinity (poor model performance). The RMSE has the power to summarize the mean difference in P and O units. Unfortunately, it could not distinguish between over- and underestimation (Jacovides and Kontoyiannis, 1995).

(2) $$RMSE=\sqrt{\frac{\sum {\left(P_{i}-O_{i}\right)}^{2}}{n}}$$

where, Oi and Pi are the observations and predictions respectively, $\bar{\text{O}}$ and $\bar{\text{P}}$represent their respective averages, and n the number of observations. Smaller RMSE value indicates more accurate simulation.

### 2.6 Baseline and alternative management practices scenarios

Alternative management practices are one of the primary strategies widely discussed by many researchers worldwide, in regard to their potential to improve the crop yield, SOC sequestration, mitigate N-leaching and GHG emissions. Smith et al. (2010) indicated that the alternative management practices are one of the most cost-effective options to mitigate GHG emissions in agriculture. The SOC is relatively dynamic and can be greatly influenced by agricultural practices.

**Figure 3:**
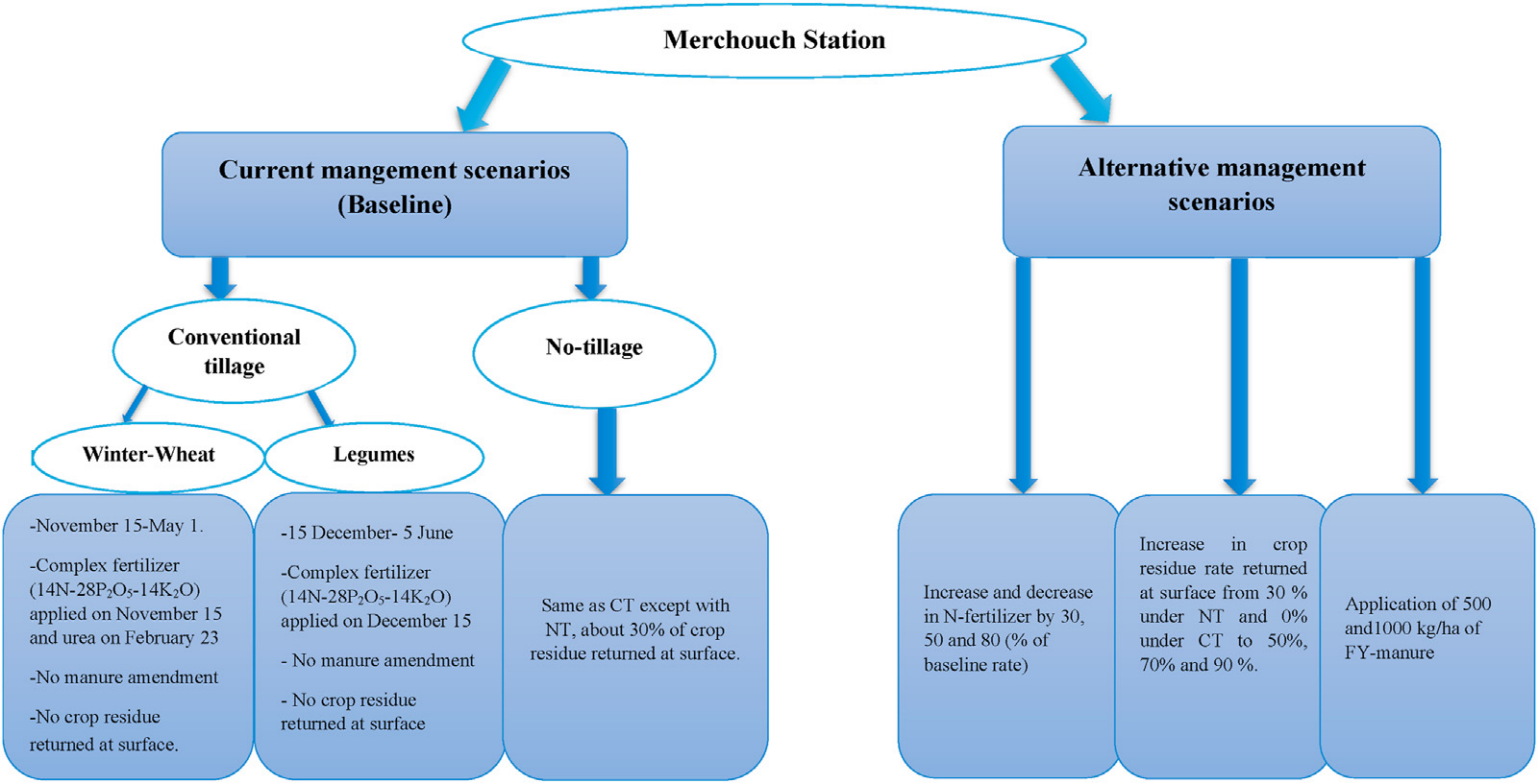
Current management practices (Baseline) and alternative management scenarios for study site, Merchouch, Morocco.

Two baseline scenarios were mainly designed for CT and NT. The two scenarios shared similar climatic conditions and soil properties. The plots received the same crop management practices (rotation, variety, seeding rate, fertilizer dose and type). Only tillage methods differentiated between the two treatments. In fact, under NT system, about 30% of crop residue was returned after harvest at surface (Figure 3).

Alternative scenarios consist of changes in crop residue returned at surface, fertilizer application rate, and manure application rate, under the two tillage practices. Practically, the alternative manure application rate was 500 or 1000 kg/ha. Alternative crop residue returned rate was 50%, 70% or 90% instead of 30% under NT, and 0% under CT (baseline rate). Moreover, the alternative fertilizer application rate was increased and decreased by 30%, 50% or 80% of the baseline fertilizer rate applied in the study site. For each simulation, only one input was varied and all other parameters remained unchanged as in baseline level.

Figure 3 summarizes the baseline and alternative management scenarios used for the simulation. DNDC was run with the baseline scenarios and all alternative scenarios for 9 years to observe their impacts on the SOC stock at Merchouch station.

SOC sequestration potential (CSP) was calculated in this work to determinate the potential of soil to sequestering SOC under each alternative scenario during the 9-simulated years. The CSP can be defined as is the difference between the average value of predicted SOC stocks under each alternative scenario (PCS), and average value of initial SOC stocks (ICS) (under baseline management). The CPS was calculated using the Equation (3) (Pathak et al., 2011). Positive and negative values refer respectively to SOC gains and losses.

(3) CSP = PCS - ICS

The unit for the three items is kg C/ha. The CSP was calculated for the two tillage practices.

## 3 Results and discussion

### 3.1 Evaluation of the DNDC model

**Figure 4:**
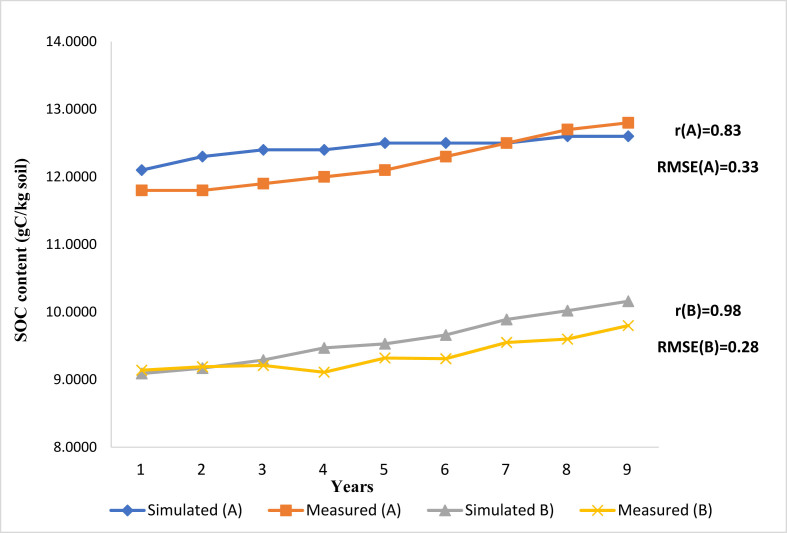
Observed and modeled SOC dynamics under no-tillage (A) and conventional tillage (B) at Merchouch Station, in 2008–2016.

The modeled results demonstrated that NT system showed a trend with an increase in SOC content compared to CT during the 9 simulated years (Figure 4). From 2008 to 2016, the average simulated SOC content was 12.5 g C/kg soil under NT, and 9.6 g C/kg soil under CT. These values were close to the ones reported by the observations (12.2 g C/kg soil, and 9.3 g C/kg soil respectively under NT and CT). On the one hand, the r between the simulated and observed SOC values was 0.83 under NT, and 0.98 under CT. On the other hand, the RMSE was 0.33 under NT, and 0.28 under CT, all< 5. The high r and low RMSE between measured and modeled values indicate that the model predictions had a good level of precision.

### 3.2 SOC contents under current management conditions

Under the baseline management conditions, modeled results showed a continuous increase in SOC content under NT during the simulated 9-years (Figure 4). An increase of 30% was observed compared to CT in the top 20 cm soil layer. This SOC improvement can be attributed to the crop residue effect, the high amount of clay, and also the low decomposition rate due to a decreased microbial activity. Further studies revealed the positive effect of NT on SOC content compared to CT after many years of practice. Moussadek et al. (2014) indicated that after five years of continuous NT system, the SOC increased by 10% in the top soil. Similarly, Laghrour et al. (2016) pointed out an increase of 30% at soil surface under NT system compared to CT after 11 years of adoption. Earlier, Mrabet, 2001 reported similar observation indicating that the SOC in Vertisol increased by 13.6% under NT after 11 years of adoption compared to CT in drier conditions. Our results are in line with other studies undertaken under semi-arid Mediterranean conditions (Lopez-Bellido et al., 1997; Murillo et al., 1998; Bescansa et al., 2006; Moreno at al., 2006; Alvaro-Fuentes et al., 2008; Lozano-Garcia and Parras-Alcantara, 2013; Fiorini et al., 2020).

Furthermore, many studies reported that SOC content increased continuously under high clay content soil compared to poor ones considering similar land use and climate conditions (Franzluebbers., 2004; Camarotto et al., 2018). A greater potential of SOC sequestration characterizes soils with fine texture such as Merchouch station. Under NT, Vertisols are “active” soils thanks to their important content on clay, leading to a significant potential of SOC stock (Lal, 1988). This clay effect is due to its stabilizing features on SOM. Indeed, Six et al. (2002) already explained the mediated aggregation process by SOC accumulation. The SOM can be trapped in very small spaces between clay particles and thus decreases the microorganism's accessibility and reduces SOC decomposition. The soil texture can disturb the soil's capacity for aggregation and adsorption, while this latter can reduce decomposition process (Krull et al., 2003).

### 3.3 Effects of crop residue and manure management practices on SOC stock

**Figure 5:**
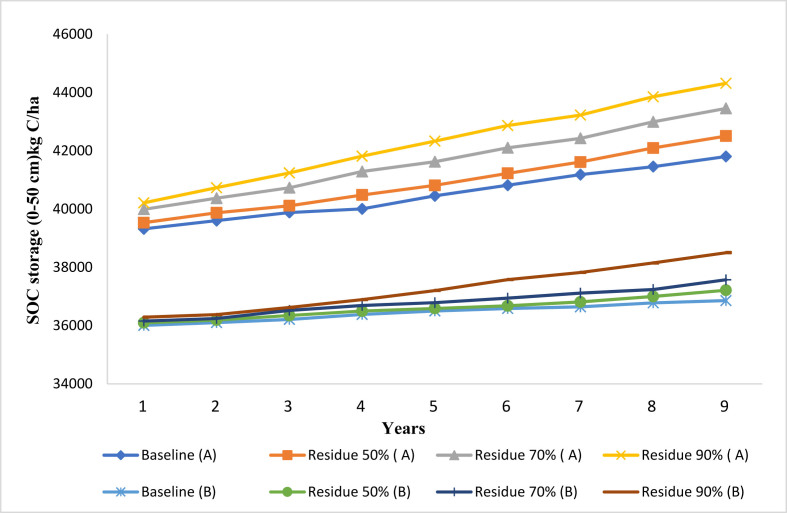
Modeled impact of alternative crop residue returned at surface on the SOC under no-tillage (A) and conventional tillage (B) system at Merchouch station.

**Figure 6:**
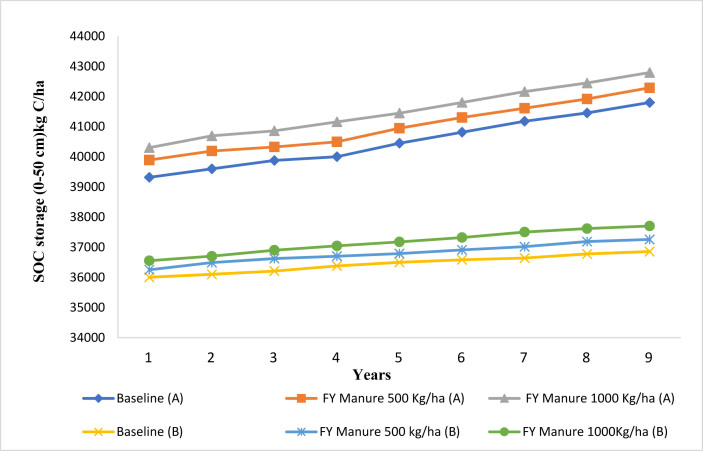
Modeled impact of manure amendment rate on SOC under no-tillage (A) and conventional tillage (B) system at Merchouch station.

**Figure 7:**
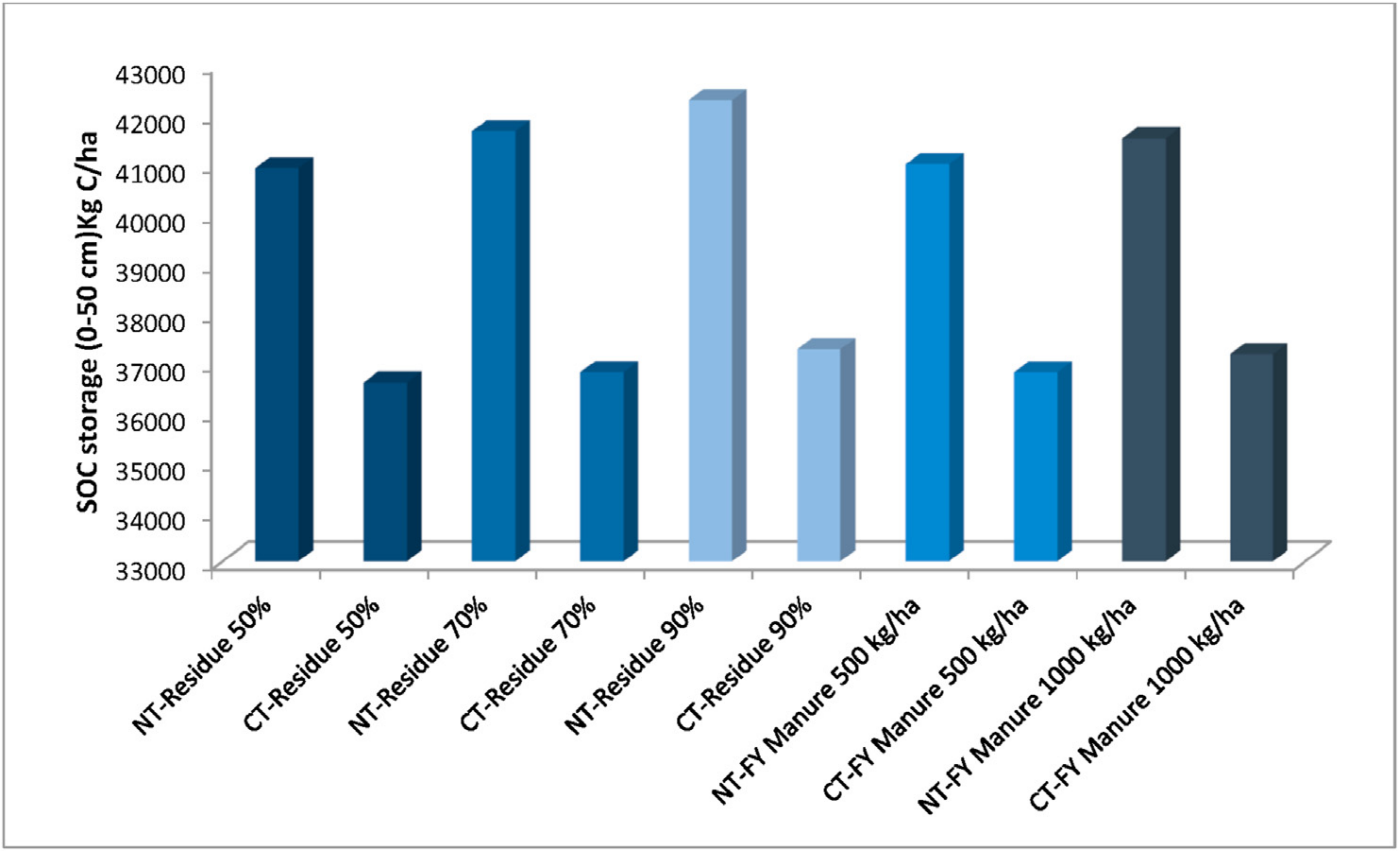
Modeled 9-year annual average of SOC stocks, under NT and CT practices, with varied crop residue rate and manure amendment.

According to the simulation results, the SOC stock successively increases across 9-years under alternative crop residue and FY-manure managements considering CT and NT practices (Figures 5 and 6). Increasing crop residue returned to the top soil layer by 50%, 70% and 90% tends to increase SOC stock by 1–4% under NT and by 0.4–0.9% under CT (Figure 5). Figure 6 exhibited modeled SOC stock under FY-manure effect and tillage practices (NT and CT). The modeled results indicated that the organic fertilizer can effectively improve the SOC stock of the study site. By increasing the FY-manure application rate from 0 (baseline) to 500 and 1000 kg/ha, the SOC increased by 1% and 3% under NT practice, and by 0.9% and 2% under CT during the period 2008–2016 (Figure 6). Additionally, CSP would range from 415 kg C/ha to 1787 kg C/ha with NT practice, and between 150 kg C/ha and 495 kg C/ha under CT system (Table 2), when the crop residue rate was modified. Furthermore, considering 500 kg/ha of farmyard manure, the CSP reached 497 kg C/ha under NT compared to 352 kg C/ha under CT system. However, CSP is estimated as 1019 kg C/ha and 818 kg C/ha respectively under NT and CT systems when adding 1000 kg/ha of FY-manure (Table 2). By comparing the impacts of crop residue and FY-manure management under NT versus CT (Figure 7), our modeled results indicated that SOC stock increased by 12–14% under alternative crop residue (50%, 70%, and 90%) under NT compared to CT. Similarly, SOC stock has been improved under NT by 11% and 12% compared to CT when adding respectively 500 and 1000 kg/ha of organic fertilizer. Our study concluded that an increase in the rate of crop residue, or application of FY-manure, especially in conjunction with the NT system, greatly increases the SOC stock through addition of SOM in the soil, and improves the capacity of soil to more sequester SOC. This finding is consistent with further studies (Li et al., 1994; Qiu et al., 2009; Kim et al., 2012; Lehtinen et al., 2014; Zhang et al., 2018; Ku et al., 2019), which revealed that application of organic fertilizer and increasing crop residue rate lead to significant enhancement of SOC stock. Similarly, Zhang et al. (2006) reported that an increase in crop residue returned from baseline management to 50% or 90%, and applying FY-manure at a rate of 500 kg/ha converted the farmland from the C source to a C sink. Tang et al. (2006), Tang et al. (2007) and Blanco-Canqui and Lal (2009) pointed out that the loss of SOC decreased by about 70% or 80%, when increasing the rate of crop residue returned from the baseline level by at least 50 %. Wang et al. (2008) attributed an increase in SOC stock with application of 1000 kg/ha of organic fertilizer, and elevating crop residue rate returned at surface after harvest from the baseline by 30%, 50% and, 80%. Moreover; Chen et al. (2018) showed that higher manure C and litter C input according to the initial manure application or crop residue used, respectively, led to the higher simulated SOC stock.

**Table 2:** DNDC modeled SOC sequestration potentials for alternative management conditions under NT and CT systems in Merchouch station- Morocco.

Alternative scenario	SOC sequestration potential (kg C/ha)	
	No-tillage	Conventional tillage
50% of crop residue returned	415	150
70% of crop residue returned	1162	355
90% of crop residue returned	1787	495
500 kg/ha of farmyard manure	497	352
1000 kg/ha of farmyard manure	1019	818
Fertilizer rate +30%∗	5	9
Fertilizer rate +50%	-14	12
Fertilizer rate +80%	20	33
Fertilizer rate - 30%	-107	-177
Fertilizer rate - 50%	-304	-269
Fertilizer rate - 80%	-535	-354

∗ % of the baseline rate.

Our results suggested that SOC stock would be substantially improved if the farmers increase the crop residue rate, applied manure fertilizer, and converted the CT to NT practice. The modeled results indicated also that the highest benefit would be gained if alternative farming management practices were applied for the poor soils containing low SOC contents especially soils in semi-arid regions. Powlson et al. (2016) and Pezzuolo et al. (2017) reported that conservation practices have the ability to increase soil fertility through increased SOC accumulation and can act as a mitigating mechanism to climate change. Previous study carried out in Nigeria (Akinde et al., 2020) indicated that the establishment of conservative soil management practices such as manuring, mulching, and conservation tillage were suggested to prevent agricultural lands from degradation by improving SOC. Our results are in line with other studies performed under semi-arid conditions (Sherrod et al., 2003; Engel et al., 2017; Sainju et al., 2017). These researchers reported that the use of improved management practices, such as NT, and increase the crop residue returned to the top soil, can curb the decline of SOC and preserve it sustainably especially in semi-arid regions.

### 3.4 Effects of fertilizer management practices on SOC stock

**Figure 8:**
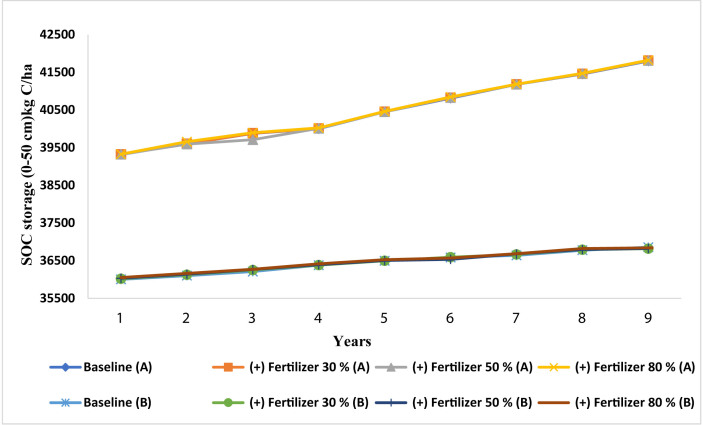
Modeled impact of increase in fertilizer rate by 30%, 50% and 80% (% of the baseline rate) on SOC under no-tillage (A) and conventional tillage (B) at Merchouch station.

**Figure 9:**
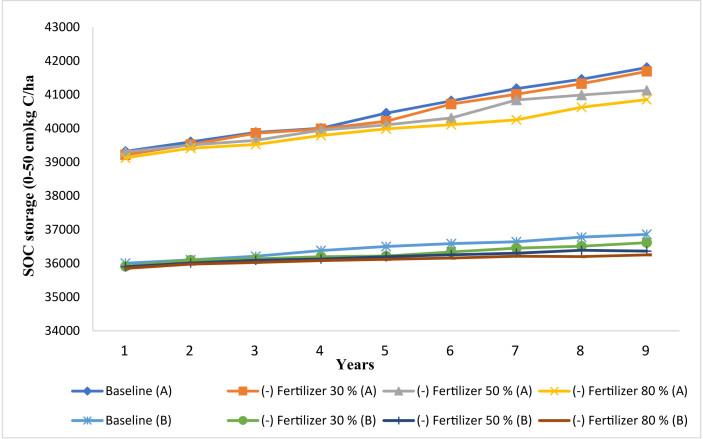
Modeled impact of decrease in fertilizer rate by 30%, 50% and 80% (% of the baseline rate) on SOC under no-tillage (A) and conventional tillage (B) at Merchouch station.

The present study aims also to evaluate the effect of change in N-fertilizer rate on SOC stock in Merchouch station. Modeled results showed that, under NT, the SOC stock increased by 0.014% and 0.04% when N-fertilizer rate was increased by 30% and 80% respectively, while SOC decreases by 0.03%, with increasing N-fertilizer rate from the baseline by 50% (Figure 8). There was a 0.02–0.09% increase in SOC stock under CT when N-fertilizer rate was increased by 30%, 50% and 80%. As shown in our simulated results, the increase in N-fertilizer rate had a negligible increase (0.014–0.09%) on SOC stock over the 9 years. Furthermore, decreasing N-fertilizer rate by 30%, 50% and 80% tends to decrease SOC stock by 0.2–1.3% under NT, while decreases by 0.4–0.9% under CT (Figure 9). According to our modeled results, CSP ranged from -14 kg C/ha to 20 kg C/ha under NT practice and from 9 kg C/ha to 33 kg C/ha under CT (Table 2) by increasing N-fertilizer rate. In contrast, CSP declined by 107–335 kg C/ha and by 177–354 kg C/ha under the NT and CT practices respectively, when decreasing N-fertilizer rate (Table 2). These results showed clearly that our study site was properly fertilized, and the rate adopted in the baseline scenarios have been reached already. Hence, further increase led to a little to negligible impact on SOC stock during the simulated years. Several studies (Farahbakhshazad et al., 2008; Wang et al., 2008; Ku et al., 2019) support our findings and confirm the absence of any considerable effect on SOC stock when increasing N-fertilization rate. Dou et al. (2014) acquired also the same results in south central USA using the DNDC model. Moreover, Segoli et al. (2013) stated that an increase in N supply, through fertilization, would stimulate SOC turnover and could decrease it. On the other hand, our results indicated that decreasing N-fertilization rate will decline SOC stock and the capacity of soil to sequester SOC. In fact, N-fertilizer rates may reduce SOC stock by increasing SOM mineralization depending on the mechanism explained by several researchers (Moorhead and Sinsabaugh, 2006; Riggs et al., 2015; Spohn, 2015; Spohn et al., 2016). These researchers demonstrate that inorganic N decreased in soil under low N-fertilizer rates conditions. At the same time, microbes hasten SOM decomposition to meet N demand for growth metabolism. This energy investment in N acquisition may reduce microbial C use efficiency. Indeed, the greatest part of decomposed SOM, from N mining, is low-or zero-energy biomolecules (such as lignin) (Spohn et al., 2016; Zang et al., 2016). This outcome is also in agreement with Sterner and Elser (2002); and Chen et al. (2014) who indicate that, if N is a limiting resource, inorganic N inputs will increase microbial biomass and activity thereby increasing SOM mineralization. Furthermore, Mulvaney et al. (2009), Russell et al. (2009), Li et al. (2017) and Mahal et al. (2019) indicated that N-fertilizer rates can led to lower SOC stocks, through the acceleration in SOM mineralization rate and decreased C sequestration efficiency in soil. In summary, our study site has already reached a good levels of N-fertilizer and hence any future change in the rates won't improve the SOC stock. Moreover, an increase in the N- fertilizer rate would be costly compared to their benefits to increase SOC stock.

## 4 Conclusion

The present study exhibited how DNDC model can be used to explicitly determine SOC stock, under different management practices and tillage systems, at Merchouch station in Morocco. The validation results showed that the DNDC model had good performance to simulate SOC stock. The simulation results indicated that: 1) the SOC content continuously increased under NT compared to CT during the 9-simulated years, 2) C additions, through an increase in crop residue rate or application of organic fertilizer, promptly increased SOC stock, especially in soils that have suffered from the degradation of SOC, 3) Merchouch station was properly fertilized, and therefore, SOC stock didn't increase significantly with increased N-fertilization rate, while the decrease in these rate effectively decreased the SOC stock. In light of these results, we can conclude that a combination of NT system with C additions through manure amendment and crop residue are the optimized management practices to improve the SOC stock especially in semi-arid areas.

## Declarations

### Author contribution statement

Ibtissame Lembaid, Rachid Moussadek, Rachid Mrabet, Ahmed Bouhaouss: Conceived and designed the experiments; Performed the experiments; Analyzed and interpreted the data; Wrote the paper.

Ahmed Douaik: Analyzed and interpreted the data; Wrote the paper.

### Funding statement

This work was supported by INRA and ICARDA corporate project.

### Data availability statement

Data will be made available on request.

### Declaration of interests statement

The authors declare no conflict of interest.

### Additional information

No additional information is available for this paper.
